# Acid-Sensing Ion Channel-1a in Articular Chondrocytes and Synovial Fibroblasts: A Novel Therapeutic Target for Rheumatoid Arthritis

**DOI:** 10.3389/fimmu.2020.580936

**Published:** 2021-01-28

**Authors:** Yayun Xu, Feihu Chen

**Affiliations:** ^1^ Department of Epidemiology and Biostatistics, School of Public Health, Anhui Medical University, Hefei, China; ^2^ The Key Laboratory of Major Autoimmune Diseases of Anhui Province, Anhui Institute of Innovative Drugs, School of Pharmacy, Anhui Medical University, Hefei, China; ^3^ The Key Laboratory of Anti-inflammatory and Immune Medicines, Ministry of Education, Hefei, China

**Keywords:** acid-sensing ion channel 1a, rheumatoid arthritis, articular chondrocyte, synovial fibroblast, therapeutic target

## Abstract

Acid-sensing ion channel 1a (ASIC1a) is a member of the extracellular H^+^-activated cation channel family. Emerging evidence has suggested that ASIC1a plays a crucial role in the pathogenesis of rheumatoid arthritis (RA). Specifically, ASIC1a could promote inflammation, synovial hyperplasia, articular cartilage, and bone destruction; these lead to the progression of RA, a chronic autoimmune disease characterized by chronic synovial inflammation and extra-articular lesions. In this review, we provided a brief overview of the molecular properties of ASIC1a, including the basic biological characteristics, tissue and cell distribution, channel blocker, and factors influencing the expression and function, and focused on the potential therapeutic targets of ASIC1a in RA and possible mechanisms of blocking ASIC1a to improve RA symptoms, such as regulation of apoptosis, autophagy, pyroptosis, and necroptosis of articular cartilage, and synovial inflammation and invasion of fibroblast-like cells in synovial tissue.

## Introduction

As a chronic systemic autoimmune disease, the main pathological characteristics of rheumatoid arthritis (RA) include synovial cell proliferation, multiple inflammatory cell infiltration, pannus formation, and cartilage and bone tissue destruction, which thereby eventually lead to joint deformity and loss of function (1). Epidemiological data show that RA affects approximately 1% of the world’s population (2). Numerous studies have demonstrated that synovial inflammation and extracellular acidification play an important role in the occurrence and development of the RA-mediated destruction of articular cartilage and bone (3, 4). Currently, the most commonly used treatment for RA is the control of synovitis rather than its root causes (5). However, the use of long-term medication reportedly cannot cure arthritis and is usually accompanied by serious side effects (6). Therefore, the key approach in preventing the progression of RA is to elucidate the pathogenesis of synovial inflammation and destruction of articular cartilage and find new targets that would control or be used to control the development of RA.

Acid-sensitive ion channel 1a (ASIC1a) is a member of the degenerin/epithelial sodium channel protein superfamily that is transiently activated by extracellular H^+^, which plays a critical role in a variety of physiological and pathological processes, including RA (7). It has been shown that extracellular acidification is a common phenomenon that plays an important role in the physiological and pathological processes related to inflammation, including early wound healing, infectious diseases, bone remodeling, and tumorigenesis (8, 9). During inflammation, the drop in pH is a result of infiltration and activation of inflammatory cells in the tissue, which leads to increased energy and oxygen demand, accelerated glucose consumption *via* glycolysis and thus increased lactic acid secretion (10, 11). In a study, decreased pH was detected in the synovial fluid of RA patients (12–14) and adjuvant arthritis (AA) rats (4), which is an animal model widely used to explore the pathophysiological mechanism of RA. Our previous studies have demonstrated that ASIC1a is involved in the injury of articular chondrocytes that is caused by increased intracellular calcium ([Ca^2+^]i) induced by extracellular acidification; this can be significantly attenuated by the use of amiloride and the ASIC1a-specific blocker psalmotoxin 1 (PCTX-1) (15–17). Recently, it has been indicated that ASIC1a is highly expressed in RA synovial tissues and RA synovial fibroblasts (RASF); it also induces synovial inflammation and invasion, which are downregulated by ASIC1a-RNAi and PCTX-1 while they are increased by the overexpression of ASIC1a (18, 19). Thus, ASIC1a may be a potential therapeutic target for RA.

In the present review, we have provided a brief overview of the molecular properties of ASIC1a and discussed the therapeutic potential of ASIC1a and the possible mechanisms of blocking ASIC1a in articular chondrocytes and synovial fibroblasts to improve the disease symptoms of RA.

## Basic Biological Characteristics of ASIC1a

ASICs are a class of extracellular H^+^-activated cation channels, also known as H^+^ non-voltage-gated cation channels, and belong to the superfamily of epithelial sodium channels (ENaC)/degradable proteins (DEG) (20). To date, seven ASIC subunits (ASIC1a, ASIC1b1, ASIC1b2, ASIC2a, ASIC2b, ASIC3, and ASIC4), which are encoded by four genes (ACCN1, ACCN2, ACCN3, and ACCN4), have been identified (21). ASICs, as acid receptors on the cell membrane, transmit the low-pH signal of the extracellular microenvironment to the cell such that the downstream signaling pathway is activated that would thereby cause a series of physiological and pathological changes (22). Compared to other ASIC subunits, ASIC1a not only has permeability to Na^+^ but also mediates the influx of extracellular Ca^2+^ (23). As a crucial secondary messenger, Ca^2+^ plays a pivotal role in the physiological and pathological processes of cells, including in RA (24). Increasing evidence indicates that ASIC1a contributes to acid-induced injury by increasing intracellular Ca^2+^ in rat articular chondrocytes and RASF.

### Structure of ASIC1a

ASIC1a is composed of more than 500 amino acids, including two hydrophobic transmembrane domains (TM1 and TM2) and one large cysteine-rich extracellular ring (25). Its N- and C-terminals are both located in the cytoplasm (26). A crystal structure analysis has revealed that ASICs exist as trimers and that three subunits are necessary for the formation of functional channels (27).

### Tissue and Cell Distribution of ASIC1a

ASIC1a is mainly distributed in the central cerebral cortex, hippocampus, cerebellum, pineal gland, amygdala, and spinal cord (28). It has been reported that ASIC1a is also expressed in isolated human monocytes and differentiated osteoclasts and is the most abundant in human chondrocytes (29). In a previous study, we found that ASIC1a, ASIC2a, and ASIC3 were expressed in the articular chondrocytes of rats with AA, and that the expression levels of ASIC1awere significantly higher than those of other subunits (4). More recently, it has been reported that the expression of ASIC1a is significantly increased in human RA synovial tissues, primary human RASF, and the ankle synovium of AA rats (18, 19). **Table 1** summarizes the tissue and cell distribution of ASIC1a.

**Table 1:** Tissue and cell distribution of ASIC1a.

Species	Tissues	Cells	References
rat, mouse, human	spinal cord	spinal dorsal horn neurons	(30)
rat, mouse, guinea pig, human	DRG, TG, NG		(31–33)
rat		astrocytes	(34)
rat		microglia	(35)
human	gliomas		(36)
rat, mouse		taste receptor cells	(37)
mouse		cortical neurons and NS20Y cells	(38, 39)
human	intervertebral disk	nucleus pulposus cells	(40)
mouse	prefrontal cortex, hippocampus		(41)
rat, rabbit	retina	cone photoreceptors, horizontal cells, some amacrine and bipolar cells	(42–44)
human	lung	epithelial cell	(45)
rat		vascular smooth muscle cells	(46)
mouse		immune cells	(47)
human, rat	bone	osteoclasts, articular cartilage	(4, 29, 48)
rat, human	synovial tissues	synovial fibroblasts	(49)
mouse		bone marrow derived macrophages, RAW 264.7 macrophage Cells	(50, 51)
mouse, human		intestinal epithelial cells, intestinal Caco-2 cells	(52, 53)
rat	urinary bladder		(54)
mouse		bone marrow-derived dendritic cells	(55)
human		monocytes	(29)
human, mouse	liver	hepatic stellate cells	(56)
hamster		Chinese hamster ovary cells	(38, 57)
rat		pulmonary arterial smooth muscle cell	(58)

DRG, Dorsal Root Ganglia; TG, Trigeminal ganglia; NG, Nodose Ganglia.

### Channel Blocker of ASIC1a

Amiloride is a potassium-sparing diuretic that regulates K^+^ and Na^+^ balance in cells. The inhibition of the ENaCs of renal tubular cells is a classic therapeutic effect of amiloride (59). There is a general consensus that amiloride also blocks ASICs, which are members of the ENaC/DEG superfamily (60). As a non-selective ASIC blocker, amiloride is one of the main pharmacological tools that is used to study the function of ASICs, including ASIC1a (61). It has been shown that amiloride and its analogs inhibit ASIC1a currents expressed in Chinese hamster ovary cells and cortical neurons (62). Among the analogs, benzamil has been reported to be the most potent ASIC1a inhibitor (62).

A-317567, a nonspecific small molecule inhibitor of ASICs, is structurally unrelated to amiloride (60, 63). A previous study has indicated that A-317567 has the potential of treating ischemic stroke due to its potent inhibition of ASIC1a-like current (60), suggesting that A-317567 has promising therapeutic potential for the treatment of ASIC1a-related diseases.

PCTX1, a peptide purified from the venom of the southern spider tarantula *Psalmopoeus cambridgei*, can specifically and strongly inhibit the current of ASIC1a (64, 65). Moreover, studies have suggested that PCTX1 can block ASIC1a in articular chondrocytes and RASF *in vitro* (4, 18, 19, 66).

Non-steroidal anti-inflammatory drugs (NSAIDs) have also been reported to inhibit ASIC1a or ASIC1a-likecurrents. For example, an *in vitro* study showed that aspirin rapidly and reversibly inhibited 83.7% of the peak ASIC current in rat cortical neurons (67). Another study has demonstrated that both ibuprofen and flurbiprofen can inhibit ASIC1a current (68), thereby indicating that the mechanism of NSAIDs in improving RA symptoms may be related to ASIC1a blockade.

A common mechanism of many local anesthetics is to block the voltage-gated Na^+^ channel (69). It has been shown that lidocaine and tetracaine can reversibly inhibit ASIC1a current (60, 70); these drugs maybe potential candidates for the treatment of ASIC1a-related diseases.

Small interfering RNA (siRNA) is a powerful tool for functional genomics and gene therapy owing to its advantages of high efficiency, specificity, and easy operation (7). In a previous study, we designed and synthesized ASIC1a-specific siRNA and transiently transfected it into rat articular chondrocytes (72). ASIC1a-siRNA could be successfully transfected into rat articular chondrocytes; after transfection, the chondrocytes reduced the expression of ASIC1a mRNA and protein, suggesting that siRNA interference technology could successfully construct the ASIC1a gene silencing cell model.

### Factors Influencing the Expression and Function of ASIC1a

As a membrane proton receptor, the number of ASIC1a present on the cell surface determines its physiological and pathological functions, and this number partially depends on protein synthesis, degradation, and membrane trafficking processes. Recently, several studies have shown that various factors affect these processes. Wang *et al.* summarized the factors regulating ASIC1a expression and activity in various conditions, including chemical regulation, metal ions, polypeptide toxins and small-molecule inhibitors, protein interactions, drugs, receptors (73). More recently, we further elucidated the major factors and underlying molecular mechanisms affecting ASIC1a protein expression and membrane trafficking (74). Specifically, acidosis, hypoxia, inflammatory cytokines, neurotrophins, hormones drugs, microRNAs, effector proteins, and chemicals have been reported to govern ASIC1a protein synthesis, degradation, and dynamic trafficking (74).

## Potential Therapeutic Targets of ASIC1a in Rheumatoid Arthritis

### Overexpression of ASIC1a in Rheumatoid Arthritis

Extracellular acidification, which promotes the defense of inflammatory cells against pathogens by regulating migration and phagocytosis, is a condition commonly associated with a variety of physiological and pathological situations (75). It has been shown that extracellular acidification is involved in the pathogenesis and development of RA (76). The pH of synovial fluid in RA patients may fall below 6.0 during active RA (12, 13, 77). Moreover, the acidification of synovial fluid has been associated with radiological joint destruction in patients with RA (14). Based on the above-mentioned studies and the fact that chondrocytes, the only cell type present in articular cartilage, are important in the pathogenesis of arthritis and are profoundly affected by local pH (78), acid-stimulated articular chondrocytes were selected as a cell model to examine the pathogenesis of RA *in vitro* (4, 66, 72, 79, 80). In previous studies, we have demonstrated that ASIC1a is expressed in rat articular chondrocytes and is increased in acid-induced chondrocytes *in vitro* (16, 81). There is growing evidence that activated RASF, a critical component of the synovial tissue to investigate the mechanism of synovial inflammation, plays a crucial role in the pathogenesis of joint destruction and RA (82). Moreover, membrane ASIC1a has been reported to be highly expressed in the RASF of three donors compared to the normal synovial fluid (NSF) of a donor (18).

Rat AA is an experimental animal model of polyarthritis and is induced by using complete Freund’s adjuvant, which is widely used as a tool to explore the pathophysiology of RA (83). In a study, a reduction in pH was detected in the synovial fluid of AA rats, and ASIC1a was upregulated in AA rat articular chondrocytes *in vivo* (4). Further studies have indicated that the expression level of ASIC1a is increased in the synovial tissues of AA rats than in normal rats, suggesting that ASIC1a may be involved in the pathological process of RA (18, 19).

Consistent with the results of studies wherein animal experiments were conducted, markedly increased expression level of ASIC1a in human RA synovial tissues with reference to those in normal synovial tissue was observed (18, 19).

### Blocking ASIC1a to Relieve Rheumatoid Arthritis Symptoms

Articular cartilage is a connective tissue located at the ends of long bones, which consists of chondrocytes and extracellular matrix (ECM) (84). Chondrocytes account for 3% of the volume of cartilage tissue and play an important role in the formation, maintenance of normal metabolism, and repair of articular cartilage (85). The ECM is mainly composed of tissue fluid, type II collagen (COII), and proteoglycan (PG) (85). The interaction between chondrocytes and the extracellular matrix maintains the normal physiological characteristics of articular cartilage (85). The degeneration of articular cartilage and the destruction of COII and PG are the most evident symptoms among arthritis-related diseases (86). Furthermore, it has been reported that AA rats treated with the ASIC1a inhibitor amiloride improved prognoses in terms of pathological articular changes (including hyperplasia of the synovium, thickening of the lining layer, formation of pannus, infiltration of a variety of inflammatory cells), and upregulation of articular cartilage matrix COII and PG (87). Moreover, the HE staining and toluidine blue staining results of pertinent cells of AA rats that received ASIC1a-specific inhibitor PCTX-1 joint injection showed that synovial invasion and cartilage destruction were ameliorated, the swelling of joint was relieved, and arthritic severity was significantly alleviated compared to those in untreated AA rats (18, 19); this indicates that ASIC1a may be a master regulator of synovial invasion and joint inflammation.

### Possible Mechanism of Blocking ASIC1a to Improve Rheumatoid Arthritis Symptoms

**Figure 1:**
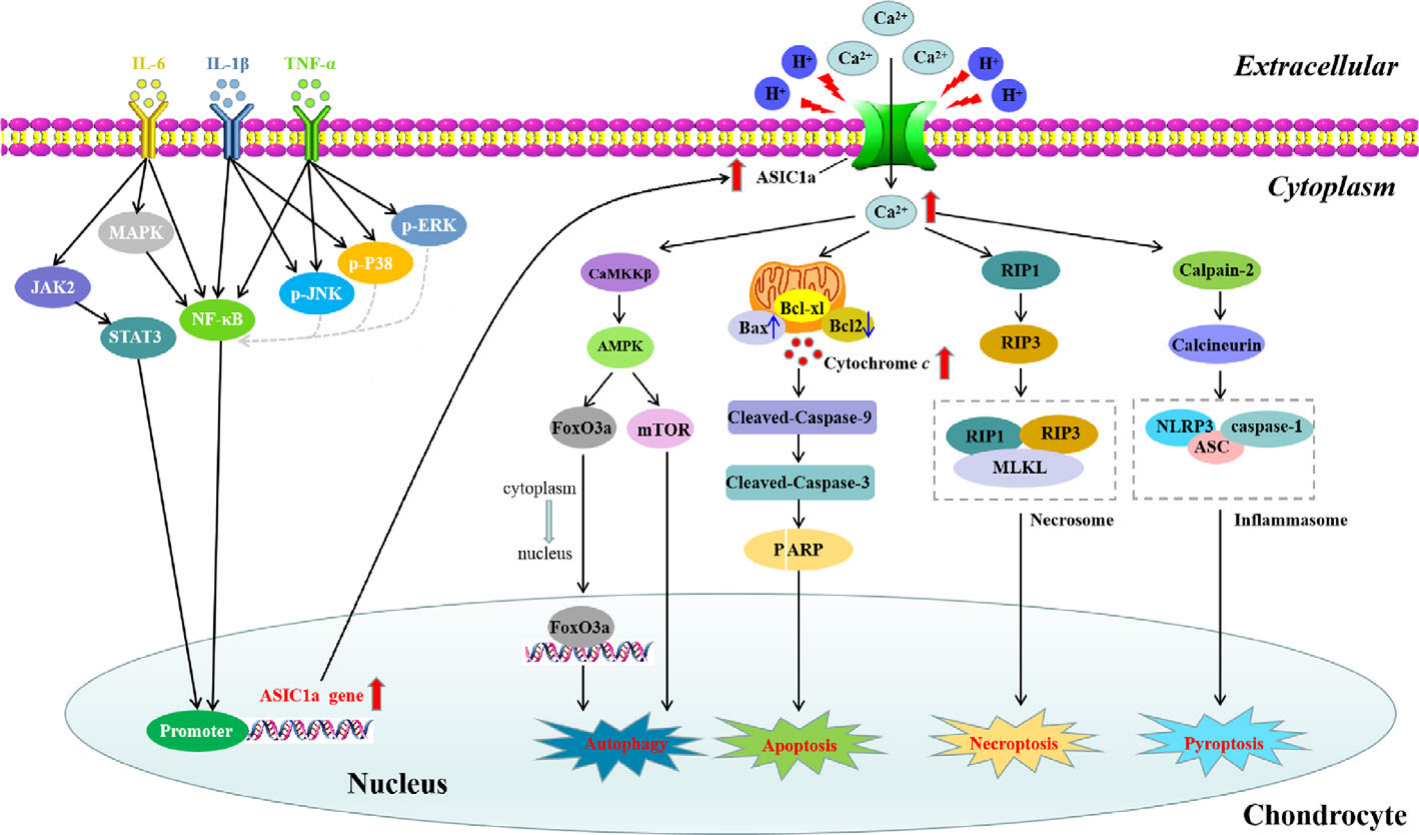
Signaling pathways involved in IL-6-, IL-1β-, and TNF-α-induced ASIC1a expression and the possible molecular mechanisms of ASC1a-mediated apoptosis, autophagy, pyroptosis, and necroptosis induced by extracellular acidification in articular chondrocytes. IL-6, IL-1β, and TNF-α activate STAT3 or/and NF-κB signaling pathways by binding to their respective receptors and lead to the upregulation of ASIC1a expression. Extracellular acidification activates ASIC1a and increases intracellular Ca^2+^ influx, which ultimately lead to the apoptosis, autophagy, pyroptosis, and necroptosis of articular chondrocytes.

**Figure 2:**
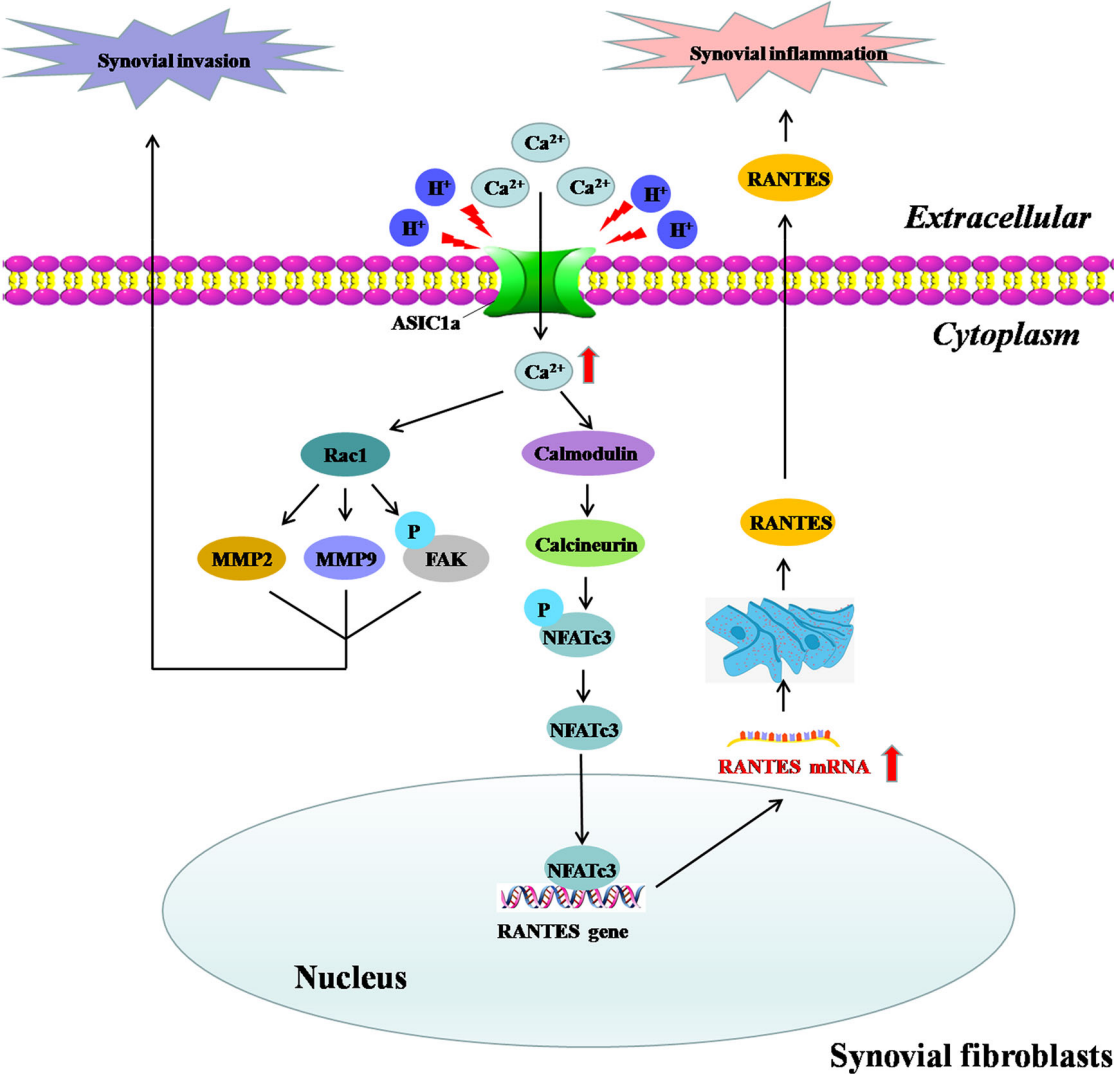
Molecular mechanism of ASIC1a-mediated synovial inflammation and invasion in RASF. Extracellular acidification activates ASIC1a and increases intracellular Ca^2+^ influx to mediate the nuclear translocation of NFATc3,which binds to the RANTES promoter to directly regulate RANTES transcription and enhance its expression in RASF; this is involved in the induction of synovial inflammation. On the other hand, ASIC1a may partially mediate the acid-induced migration and invasion of RASF through [Ca^2+^]i-Rac1 signaling, thereby contributing to the synovial invasive destruction of cartilage.

ASIC1a is expressed in human and rat articular cartilage and synovium; it is also upregulated during acid induction and inflammatory state, which are the two main initiating factors in the occurrence and development of RA (4, 18, 19). In this section, we explore the mechanism by which ASIC1a improves RA symptoms and leads to the alleviation of the destruction of articular cartilage (**Figure 1**) and synovial inflammation and invasion (**Figure 2**).

#### ASIC1a Is Involved in the Regulation of Apoptosis in Articular Cartilage in RA

Apoptosis, which is an autonomic ordered programmed cell death, plays a significant role in normal homeostasis, physiologic cell removal, and inflammatory joint diseases occurring in RA (88). Current evidence indicates that the early and late stages of apoptosis of primary human chondrocytes contribute to the joint damage observed in the pathogenesis of RA (89). In a previous study, we showed that more than half of the chondrocytes underwent apoptosis after extracellular acidification (15). Treatment with amiloride in acid-induced chondrocytes resulted in a dose-dependent decline in apoptosis (15). More specifically, amiloride partly restored mitochondrial membrane potential by regulating the mRNA expression of apoptosis-related Bcl-2 family genes and caspase 3/9 activity in chondrocytes induced by extracellular acid (15). Another study also demonstrated that blocking ASIC1a by amiloride could protect articular chondrocytes from acid-induced apoptosis through the downregulation of Ca^2+^-dependent signaling pathways such as calpain and calcineurin and the inhibition of caspase-3 activity (17); these findings indicated that ASIC1a mediated the apoptosis of acid-induced articular chondrocyte by causing [Ca^2+^]i overload.

Proinflammatory cytokines such as interleukin-1β (IL-1β) and tumor necrosis factor-α (TNF-α) lead to the progressive destruction of articular structures in RA by stimulating synovial hyperplasia and bone destruction (90). A recent study reports that extracellular acidosis activates ASIC1a and reduces cell viability by triggering the apoptosis of rat articular chondrocytes and that pretreatment with TNF-α and IL-1β can enhance this process (4). Furthermore, IL-1β and TNF-α upregulated the expression of ASIC1a in primary rat articular chondrocytes in a time- and dose-dependent manner. Moreover, pretreatment with IL-1β and TNF-α significantly reduced cell viability and increased LDH release, [Ca^2+^]i elevation, and apoptosis by promoting the depolarization of the mitochondrial membrane and by upregulating the expression levels of cleaved PARP, cleaved caspase-3, and cleaved caspase-9 in acid-induced articular chondrocytes; the blockade of ASIC1a with PcTX1 or ASIC1a-shRNA dramatically decreased the expression of pro-apoptotic proteins (4). These results indicated that the pro-apoptotic effects of IL-1β and TNF-α in acid-stimulated articular chondrocytes are at least partially due to their involvement in regulating the expression and function of ASIC1a.

#### ASIC1a Is Involved in the Regulation of Autophagy in Articular Cartilage in RA

As an intracellular degradation system, autophagy mainly promotes the degradation of long-lived proteins and provides nutrition for the survival of cells during starvation (91). Increasing evidence shows that autophagy may lead to the degradation of damaged or organelles in excess, including mitochondria and endoplasmic reticulum (92). Recently, autophagy has been found to not only play a role in cell protection but also in the promotion of cell death in many types of cells. The overactivation of autophagy can lead to type II programmed cell death (93). In a study, it has been demonstrated that the level of autophagy in the synovial tissue of patients with active RA is increased and is related to the severity of the disease, thereby indicating that autophagy may play a crucial role in the regulation of RA (94). Intracellular Ca^2+^ is a regulator of autophagy (95). In a previous study, we showed that acidified activated ASIC1a promoted the autophagy of articular chondrocytes by mediating extracellular Ca^2+^ influx (96). Extracellular acidification could increase the levels of ASIC1a and autophagy-related markers (including LC3B-II and Beclin1) in articular chondrocytes, which was inhibited by the ASIC1a-specific blocker PcTx1 and the calcium-chelating agent BAPTA-AM (96). Moreover, the AMPK/FOXO3a signaling pathway has been considered to be involved in ASIC1a mediated autophagy. Extracellular acidification could activate AMPK and increase the levels of total FOXO3a and intranuclear FOXO3a, which could be reversed by the blockage of ASIC1a with PcTx1 or the blockage of Ca^2+^ with BAPTA-AM (96). The gene silencing of AMPK and FOXO3a can reduce the expression of LC3B-II and other autophagy-related markers in acid-induced articular chondrocytes (96).

Ca^2+^/CaMKKβ/AMPK/mTOR pathway has been reported to be involved in the progression of autophagy (97). Thus, the CaMKKβ/AMPK/mTOR signaling pathway was also evaluated to examine the mechanisms of ASIC1a in autophagy. It has been shown that the downregulated protein levels of p-mTOR/mTOR and the upregulated protein levels of CaMKKβ/β-actin and p-AMPK/AMPK in acid-induced activated articular chondrocytes were reversed by the inhibition of ASIC1a or BAPTA-AM; this suggests that the CaMKKβ/AMPK/mTOR signaling pathway may be related to the role of ASIC1a in autophagy (72).

Altogether, these results suggest that Ca^2+^ is an important factor in the acid-induced autophagy of articular chondrocytes and that ASIC1amayact as an upstream regulator of autophagy by inhibiting the influx of intracellular Ca^2+^.

#### ASIC1a Is Involved in the Regulation of Pyroptosis in Articular Cartilage in RA

Pyroptosis, which is a proinflammatory programmed cell death, is characterized by caspase-1 activation and the secretion of the proinflammatory cytokines IL-1β and IL-18 (98). Pyroptosis is inherently inflammatory and involves the activation of a caspase-activating complex known as the inflammasome (99). The inflammasome is mainly composed of three parts: receptor proteins, apoptosis-associated speck-like protein (ASC), and effect molecule pro-caspase-1 (100). According to different receptor proteins, inflammasomes can be divided into NLRP1 (101), NLRP3 (102), NLRC4 (103), and AIM2 inflammasome (104). Recent studies have found that pyroptosis is related to the occurrence and development of some autoimmune diseases, such as RA (105, 106). In cells exposed to Ca^2+^, bacterial toxin, and H^+^, the classical signaling pathway of the pyroptosis mediated by caspase-1 is activated (107, 108). Thus, in our previous study, we aimed to observe the pyroptosis of articular chondrocytes during the occurrence of RA *in vivo* and *in vitro* and to explore the role of ASIC1a in the pyroptosis of articular chondrocytes and its possible mechanism (87). The results of the *in vivo* experiments showed that ASC, NLRP3, caspase-1, IL-1β, and IL-18 were upregulated in the joints of AA rats compared to those in normal rats, whereas the expression of Col2a in cartilage was decreased. These changes were reversed by the ASIC1a inhibitor amiloride (87). Consistently, the results of *in vitro* experiments showed that the expression levels of ASC, NLRP3, caspase-1, IL-1β, and IL-18 were increased by extracellular acidosis and were reversed by PCTX-1 or BAPTA-AM (87). These results indicate that ASIC1a mediates pyroptosis in chondrocytes from AA rats, a mechanism which may be associated with the ability of ASIC1a to promote [Ca^2+^]i and upregulate the expression of the NLRP3 inflammasome.

#### ASIC1a Is Involved in the Regulation of Necroptosis in Articular Cartilage in RA

Necroptosis, a necrotic cell death pathway regulated by receptor interacting protein (RIP) 1 and 3, plays a critical role in the pathogenesis of several human inflammatory diseases (109). A recent study has shown that leptin can protect chondrocytes from necrosis, thereby indicating that necrosis may be related to the death of chondrocytes (110). The serine/threonine kinase activity of RIP1 is essential for necroptosis (111). Moreover, it has been reported that acid stimulation recruits RIP1 to the C-terminus of the ASIC1a in the cytoplasm; this causes the phosphorylation of RIP1 and subsequent neuronal death (112). Moreover, the deletion of ASIC1a gene significantly prevented the phosphorylation of RIP1 and brain injury, thereby indicating that the activation of RIP1 mediated by ASIC1a played an important role in ischemic neuronal injury (112). In a study, ASIC1a and RIP1 immunostaining signals were found to be highly co-expressed in articular cartilage tissues of AA rats than in control rats by using double-labeling immunofluorescence (79). Necrostatin-1 is a potent inhibitor of RIP1 kinase activity (113). In a previous study, we demonstrated that necrostatin-1 could reduce articular cartilage damage and necroinflammation in AA rats (79). Additionally, the ASIC1a-specific blockers PcTX-1 or ASIC1a short hairpin RNA inhibited the increase in the acid-induced necrosis markers RIP1 and RIP3, respectively, suggesting that acid-induced chondrocyte necrosis was mediated by ASIC1a (79). These findings indicate that blocking the ASIC1a-mediated necrosis of chondrocytes may provide a potential therapeutic strategy for the treatment of RA.

#### ASIC1a Promotes Synovial Inflammation and Invasion of Fibroblast-Like Cells in RA

Several studies have shown that fibroblast-like cells (FLSs) contribute significantly to the initiation and perpetuation of RA (114). Specifically, it has been reported that stable activated FLSs of RA can escape the growth limit by contact inhibition and gain the ability to migrate and invade, thereby leading to RA progression and cartilage destruction (115). Therefore, inhibiting the migration and invasion of RA-FLSs maybe a therapeutic strategy for the destructive progression of RA (116). Previous studies have suggested that ASIC1amaybe related to the proliferation and migration of tumors (117, 118). We explored the role of ASIC1a in the migration and invasion of RA-FLSs and whether blocking ASIC1a could reduce the migration and invasion of RA-FLSs and control the progression of RA (19). The results of HE and toluidine blue staining showed that synovial invasion was inhibited by the ASIC1a-specific inhibitor PcTX-1 in AA rats (19), thereby indicating that the inhibition of ASIC1a protected articular cartilage from synovial invasion and destruction in RA. Furthermore, the possible mechanism of ASIC1a involvement in synovial invasion was explored. As the key members of the MMP family, MMP-2, and MMP-9 have the ability to cleave gelatin, collagen type I, IV, and V, elastin, and vitronectin, which provide the conditions for cell migration and invasion (119, 120). Focal adhesion kinase (FAK), a non-receptor protein tyrosine kinase, plays a key role in integrin-mediated signaling pathways that are relevant to cell adhesion, migration, and invasion (121). ASIC1a-RNAi and PCTX-1 have been reported to decrease the extracellular acidification-induced invasion and migration of RA-FLSs and the expression of MMP2, MMP9, and p-FAK, which are upregulated by ASIC1a (19), suggesting that these proteins may be downstream signaling molecules of ASIC1a that are involved in synovial migration and invasion. Additionally, Ras-related C3 botulinum toxin substrate 1 (Rac1), a member of the Rho family of Ras-like small GTPases, interacts with a series of effectors and thus mediates various biological functions (122). Ca^2+^/Rac1 signaling has been reported to regulate the migration and invasion of RA-FLSs (123, 124). In a study, we demonstrated that the migration, invasion, and expression of MMP2, MMP9, and p-FAKin RA-FLSs were decreased by the intracellular calcium chelating agent BAPTA-AM or Rac1 specific blocker NSC23766 (19).Thus, we conclude that ASIC1a maybe a master regulator of synovial invasion *via* the Ca^2+^/Rac1 pathway and that inhibition of synovium invasion maybe one of the mechanisms forASIC1a in the treatment of RA.

Synovial inflammation is the main pathogenic factor of RA and plays an important role in the occurrence and development of RA-mediated articular cartilage and destruction of the bone (125, 126). RASF, as a key cell component of synovial tissues, plays an important role in synovial inflammation and joint structure destruction (76). The acidification of synovial fluid may be a key factor in synovial inflammation during the pathogenesis of RA (127). In a study, we demonstrated that extracellular acidification induced cartilage destruction by activating ASIC1a (87). For synovial tissues, it has been shown that the expression of ASIC1a in human RA synovial tissue, primary human RASF, and ankle synovium of AA rats was significantly increased (18). Consistently, *in vitro* experiments showed that extracellular acidification upregulated the expression of ASIC1a in RASF. Moreover, the expression of the inflammatory cytokines “regulated on activation, normal T cell expressed and secreted” (RANTES), sTNF RI, MIP-1a, IL-8, sTNFRII, and ICAM-1 were increased in RASF by extracellular acidification, which was significantly attenuated by ASIC1a-RNAi and PCTX-1. This indicated that ASIC1a induced synovial inflammation. Nuclear factor of activated T cells (NFATs), as a group of calcium-dependent transcription factors, regulate the transcription of inflammation-related genes, which drive the inflammatory process (128). Emerging evidence has indicated that ASIC1a induces the nuclear translocation of NFAT3 by mediating Ca^2+^ into pulmonary artery smooth muscle cells (129). Our research showed that extracellular acidification activated ASIC1a for the mediation of [Ca^2+^]i and the nuclear translocation of NFATc3. RANTES, also known as chemokine C-C motif ligand 5 (CCL5), is an important inflammatory cytokine that promotes the progression of RA. Recent studies have shown that the levels of RANTES in the plasma, synovial fluid and synovial tissue of RA patients are increased (130). NFATc3 binds to the RANTES promoter, directly regulates the transcription of RANTES, and enhances its expression; thus, RANTES is involved in the induction of synovial inflammation. These findings suggest that ASIC1a may be a potential therapeutic target for preventing synovial inflammation and controlling the progression of RA.

Acid-base balance is an important condition for maintaining normal physiological activities. Many diseases, such as ischemia, inflammation, hypoxia, and cancer, cause pH changes. ASICs, a member of the extracellular H^+^-activated cation channel family, affects the pathological and physiological changes of tissues. Numerous evidences have shown that ASIC1a promotes inflammation, synovial hyperplasia, articular cartilage, and bone destruction, which together lead to the progression of RA; these pathologic events maybe significantly attenuated by blocking ASIC1a, thereby indicating that ASIC1a may represent a novel target for the treatment of RA. The mechanism of ASIC1a blockade to improve symptoms of RA may be related to reduced apoptosis, autophagy, pyroptosis, and necroptosis of articular chondrocytes and inflammation and invasion of synovium. Further studies are required, especially in humans, to comprehensively explore the therapeutic potential of ASIC1a in RA.

## Author Contributions

YX and FC designed this work and revised the manuscript. YX wrote the manuscript. All authors contributed to the article and approved the submitted version.

## Funding

This work was supported by the National Natural Science Foundation of China (grant number 81873986).

## Conflict of Interest

The authors declare that the research was conducted in the absence of any commercial or financial relationships that could be construed as a potential conflict of interest.
